# Hidradenitis suppurativa and female infertility: a pilot study conducted amongst 110 dermatological patients

**DOI:** 10.1007/s00403-024-03390-6

**Published:** 2024-09-28

**Authors:** Cecilia Egede Medianfar, Christoffer Kursawe Larsen, Sara Karoline Saunte, Ditte Marie Louise Saunte, Gregor Borut Ernst Jemec, Rune Kjærsgaard Andersen

**Affiliations:** 1 Department of Dermato-Venereology, Zealand University Hospital, Roskilde, Denmark; 2 Department of Clinical Medicine, Faculty of Health Science, University of Copenhagen, Copenhagen, Denmark; 3 Department of Immunology and Microbiology, University of Copenhagen, Copenhagen, Denmark; 4 Department of Dermatology, Zealand University Hospital is a part of the European Reference Network On Rare and Undiagnosed Skin Disorders, Sygehusvej 10, 4000 Roskilde, Denmark

**Keywords:** Hidradenitis suppurativa, Infertility, Fertility, PCOS, Pregnancy

## Abstract

Hidradenitis suppurativa (HS) is a chronic inflammatory skin disease with severe consequences on quality of life. One aspect of this is that HS has been linked to an increased risk of infertility, but as data is still limited, it is not known if the association reflects causality. We performed a cross-sectional study of 161 female participants included at Zealand University Hospital, Roskilde, Denmark. Data regarding basic demographics, infertility, pregnancies, sexual function, and known factors with impact on fertility was collected. Logistic regressions were performed to analyze the association between HS and infertility, adjusting for factors that influence fertility. The study population consisted of 55 patients with HS, 55 patients with other dermatological diseases (ODD), and 51 healthy controls (HC). The HS group had a higher BMI, lower sexual function, and a higher tobacco use. We found no significant difference between the three groups in terms of whether they had children, the number of children they had, infertility, or whether they had received fertility treatments. The logistic analyses found no correlation between HS and infertility, and adjusting for factors affecting infertility did not alter this result. This study did not find a significant difference in infertility between patients with HS compared to those with ODD or HC. This result was not affected by adjustments for other factors known to influence fertility. Interestingly, the lower sexual function in the HS group did not appear to influence their desire or ability to conceive.

**Supplementary Information:**

The online version contains supplementary material available at 10.1007/s00403-024-03390-6.

## Introduction

Hidradenitis suppurativa (HS) is an inflammatory skin disease characterized by the formation of painful nodules, abscesses, and skin tunnels, primarily affecting intertriginous areas such as the axilla, groin and perigenital region [1–3]. HS has an estimated prevalence of 0.7–1.2% in the European-US population [1, 2]. HS has severe social and interpersonal consequences, including a significant decrease in quality of life, sexual dysfunction, an increased risk of developing mental health disorders, and of completed suicide [1, 2].

The pathophysiology of HS is only partially understood, but systemic inflammation is among the key factors [1, 2]. The role of sex hormones in HS pathogenesis is unclear [1], though findings of menstrual cycle-associated flare-ups [1, 3] and sex-dependent differences in lesion location [3] suggest a correlation. Data analyzing total free androgen levels in patients with HS are contradictory, with one study finding increased concentrations [4], while another shows no significant variation when compared to healthy controls [5]. Increased expression of androgen receptors in affected skin may explain these findings [3]. This is supported by reports of decreased lesion count and pain scores when treated with anti-androgenic medications [1, 6], and disease worsening with the use of oral contraceptives containing pro-androgenic progestogens [7].

HS is associated with a high burden of comorbidities, including obesity, metabolic syndrome, and arthritis [8]. Of particular interest in the context of fertility, HS is associated with polycystic ovarian syndrome (PCOS) [1, 2, 9], which in turn is associated with infertility [10, 11]. A main component of PCOS is hyperandrogenism [12, 13]. Dysregulation in androgen levels or androgen susceptibility, as seen in both PCOS and HS, could constitute a potential pathway through which HS patients may experience an increased risk of infertility.

However, information on HS and infertility is scarce. Adverse pregnancy and childbirth outcomes—such as the higher risk of abortions, cesarean delivery, and lower odds of live birth [14], decreased fulfillment of reproductive desires, and decreased desire for pregnancy [15], are amongst reported findings. Infertility has been linked with HS in a subset of patients [16, 17], but these findings are limited. To our knowledge, studies aiming to isolate the effect of HS on fertility, adjusting for confounding factors known to increases infertility, have not been performed.

The aim of this study is therefore to explore a connection between HS and infertility, taking into account other factors, such as the presence of PCOS, which could influence the fertility of the patients.

## Methods

We conducted a cross-sectional study examining the association between HS and infertility, adjusting for other factors known to influence fertility. Patients with HS, patients with other dermatological diseases (ODD), and healthy controls (HC) completed questionnaires regarding, among other things, pregnancy and menstrual history, factors with influence on fertility, and sexual function.

### Inclusion and exclusion criteria

Inclusion criteria were female sex, the ability to read and understand Danish, and being over 18 years of age. The exclusion criteria were the inability to participate due to a severe psychiatric disorder or being under the guardianship of others.

**Fig. 1:**
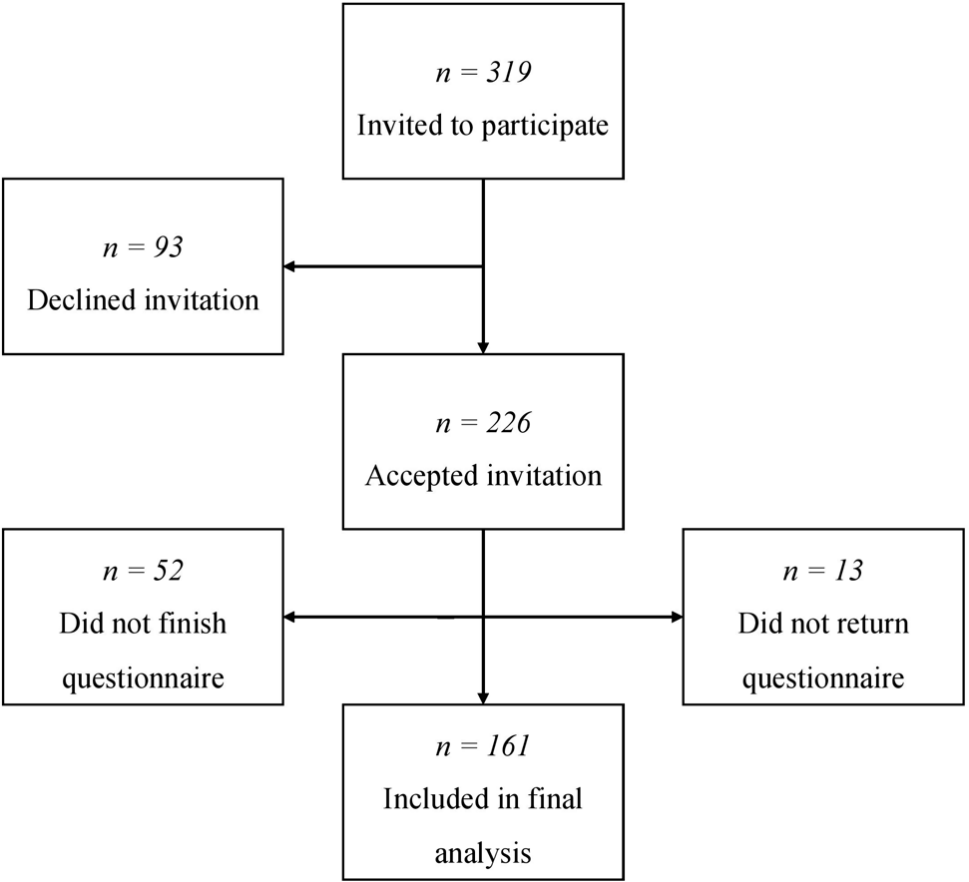
Inclusion flowchart

Inclusion was conducted from September 2019 to November 2021, with the extended collection period owing to the COVID-19 lockdown, during which non-essential activities were temporarily put on hold. A total of 319 individuals were invited to participate. Of these, 93 declined the invitation. Of the 226 participants, 52 did not finish, and 13 failed to return the questionnaire. Consequently, 161 participants were included in the final analysis (Fig. 1).

Both patient groups were recruited at the Dept. of Dermatology, Zealand University Hospital, Roskilde, Denmark. The HC group consisted of companions to patients seen at the Dept. of Dermatology and a small number (8 persons) of auxiliary staff at the Dept. of Pathology Zealand University Hospital, Roskilde.

### Questionnaire

Data aimed at evaluating fertility were obtained via a simple questionnaire. We collected information on basic demographics, the numbers of pregnancies, childbirths, or stillborn/abortions (spontaneous/provoked), whether the participants attempted pregnancy, the length of time they tried, and whether IVF treatments were attempted.

Factors known to influence fertility were also collected. These included medical and surgical procedures (e.g. methotrexate, surgery on reproductive organs), environmental factors (e.g. smoking in pack years, alcohol in orderly groups of units of alcohol per week), and diseases (e.g. PCOS). These factors were later grouped into four categories: diseases and symptoms before pregnancy, treatments before pregnancy, treatments during pregnancy, and male partner comorbidities before pregnancy (see Supplementary Material for an exhaustive list). All participants were asked to provide answers for exposures before, during, and after pregnancy. Participants who had not conceived provided answers for the time before, during, and after their 30th year of life. This time point was chosen for easy comparison, as the median age of first-time birth in Denmark is 30 years [18].

Information about sexual function was measured using the Female Sexual Function Index (FSFI) [19, 20]. It was included in the analysis to assess the role of sexual function as a possible confounder. The FSFI is a validated questionnaire consisting of 19 items regarding sexual stimulation and behavior. It contains questions about desire, arousal, lubrication, orgasm, satisfaction, and pain, all scored on a range from 0 (or 1) to 5. The minimum overall score is 2.0 and the maximum score is 36.0, with higher scores indicating higher levels of sexual function. The participants were asked to respond according to their status in the period they were trying to get pregnant, at age 30, or current age if younger than 30.

Data on HS in the patient group included age at diagnosis, affected areas, visual analog scale (VAS) of disease severity, and whether the patients refrained from sex due to pain. In the ODD group, patients were inquired about their disease, whether they were diagnosed before, at, or after 30 years of age, disease severity measured by VAS, and whether the disease led to refraining from sex. The HC group was asked to confirm that they did not suffer from any dermatological disease.

We calculated the fertile period of the participants as the years from menarche to menopause, excluding years with contraceptive use, and infertility was defined by the World Health Organization (WHO) as unsuccessfully attempting to become pregnant for more than 12 months [21].

### Statistical analysis

For quality control, all data were entered twice into a predefined Excel spreadsheet. Statistical analysis was performed using R-software. For continuous values, results are provided as either means and standard deviations or medians and interquartile ranges. Variance was measured via the ANOVA test or the Kruskal–Wallis test, depending on normality. If a significant difference in variance between the groups was found, a Tukey Post Hoc analysis or a post-hoc Dunn’s Test, respectively, was performed. For categorical values, a Chi-square-test was performed to evaluate variance. If a significant result was found, a Pairwise Comparison post-hoc test was performed. Any correlation between the factors with influence on fertility was assessed via Spearman’s test. To avoid over-adjusting of the multivariate analysis, it was decided that for variables with a correlation of *ρ* > 0.5, only one of them could be used in the subsequent adjusted models. The only variables that possessed such a correlation were “diseases and symptoms during pregnancy” and “diseases and symptoms before pregnancy” (*ρ* = 0.59, *p* < 0.001). Therefore, diseases and symptoms during pregnancy were excluded from the analysis described below.

Univariate and multivariate analyses were performed as logistic models with the outcome of infertility, adjusting for the different factors with influence on fertility. For the adjusted models, only participants who had specified to either having had children, had been pregnant, or had attempted to conceive were included. Three logistic models were constructed to assess the effect of HS on infertility. Model 1 was adjusted for the effects of age and BMI at the first attempt of conception. Model 2 was additionally adjusted for the effects of tobacco, alcohol, the duration of the fertile period, and sexual function. Finally, model 3 was additionally adjusted for the effects of FSFI-score, HS severity, ODD severity, diseases before pregnancy, treatments before pregnancy, treatment during pregnancy, and male partner comorbidities before pregnancy. *p* values under 0.05 were regarded as significant. To counteract multiple testing, the Bonferroni correction was applied.

## Results

### Basic demographics

Of the 161 participants included in this study, 55 were patients with HS, 55 were patients with ODD, and 51 were HC. The three groups differed in BMI at pregnancy/30 years of age, with the HS group having a higher median BMI compared to the ODD group (*p* = 0.01). We also observed a higher median BMI at inclusion for the HS group compared to both the ODD (*p* = 0.0007) and HC (*p* = 0.005) groups. Additionally, the HS group has higher median pack years before pregnancy compared to both the ODD (*p* = 0.007) and HC (*p* = 0.001) groups. Furthermore, the self-evaluated disease severity was significantly higher for the HS group compared to the ODD group (*p* = 0.0002). No statistical difference was found in age at the time of inclusion (*p* = 0.347), marital status (*p* = 0.276), the distribution of participants within each group regarding whether they had children or not (*p* = 0.19), or alcohol intake (*p* = 0.3611). See Table 1 for full details on demographics.

**Table 1:** Basic demographics

	HS (*n* = 55)	ODD (*n* = 55)	Healthy (*n* = 51)	*p* value
Age at inclusion				
Mean (SD)	42.4 (12.14)	41.3 (12.10)	45.1 (14.68)	0.347
N/A	0	0	0	
Marital status				
Married *n* (%)	16 (29.1)	21 (38.2)	26 (51.0)	0.276
Cohabiting *n* (%)	20 (36.4)	19 (34.5)	11 (21.6)	
Relationship *n* (%)	10 (18.2)	10 (18.2)	9 (17.6)	
Single *n* (%)	9 (16.3)	5 (9.1)	4 (7.8)	
N/A	0 (0)	0 (0)	1 (2.0)	
BMI at pregnancy/30 years of age				
Median (IQR)	25.4^†^ (22.4; 29.9)	22.9^†^ (20.4; 25.2)	23.9 (21.7; 27.8)	**0.02**
N/A	4	4	4	
BMI at inclusion				
Mean (SD)	33.6^†,‡^ (0.32)	27.8^†^ (0.29)	28.6^‡^ (0.33)	**0.0004**
N/A	6	5	6	
Has given birth to at least one child				
Yes *n* (%)	37 (67.3)	39 (70.9)	42 (82.4)	0.190
No *n* (%)	18 (32.7)	16 (29.1)	9 (17.6)	
N/A	0	0	0	
Smoking (pack years)				
Before pregnancy median [IQR]	6.0^†,‡^ (0; 12.38)	0^†^ (0; 4.63)	0^‡^ (0–5.06)	**0.0007**
*Alcohol (units)*				
Before pregnancy *n* (%)				
0/week	29 (52.7)	30 (54.5)	24 (47.1)	0.3611
1–7/week	20 (36.4)	23 (41.9)	26 (51.0)	
8–14/week	5 (9.1)	2 (3.6)	1 (1.9)	
15–21/week	1 (1.8)	0 (0)	0 (0)	
Disease severity, VAS (transformed to score from 0 to 100)				
Median (IQR)	20.0^†^ (0; 60.0)	0^†^ (0; 12.5)		**0.0002**

Significant *p*-values are highlighted in bold

*BMI* body mass index, *HS* hidradenitis suppurativa, *IQR* interquartile range, *N/A* not available, *ODD* other dermatological diseases, *SD* standard deviation, *VAS* visual analog scale

^†^ Indicates significant difference between the HS group and the ODD group in a post hoc analysis in BMI at pregnancy (*p* = 0.02), BMI at inclusion (*p* = 0.0007), and smoking (*p* = 0.007)

^‡^ Indicates significant difference between the HS group and the healthy group in a post hoc analysis in BMI at inclusion (*p* = 0.005) and smoking (*p* = 0.001)

### Pregnancies and infertility

According to the WHO definition of infertility, 25.5% of the HS group, 18.2% of the ODD group, and 15.7% of the HC group were classified as infertile (*p* = 0.42). The lifetime median (IQR) number of pregnancies was similar across the groups (*p* = 0.25). No significant difference was found in the percentage of participants who had received fertility treatment (*p* = 0.33). The FSFI (median [IQR]) was found to be lower for the HS group (22.4 [5.6; 31.6]) compared to the HC group (28.9 [22.2; 32.9]) (*p* = 0.03) (see Table 2).

**Table 2:** Pregnancies and infertility

	HS (*n* = 55)	ODD (*n* = 55)	Healthy (*n* = 51)	*p* value
Infertility (Unsuccessfully attempted to conceive > 12 months)				
Yes *n* (%)	14 (25.5)	10 (18.2)	8 (15.7)	0.42
No *n* (%)	41 (74.5)	45 (81.8)	43 (84.3)	
Number of pregnancies				
Median (IQR)	2 (1.0; 2.0)	2 (1.0; 3.0)	2 (1.0; 3.0)	0.25
IVF				
Yes *n* (%)	8 (14.5)	7 (12.7)	3 (5.9)	0.33
No *n* (%)	47 (85.5)	48 (87.3)	48 (94.1)	
Number of abortions				
Median (IQR)	0 (0; 1)	0 (0; 1)	0 (0; 1)	0.89
FSFI-score				
Median (IQR)	22.4 (5.6; 31.6)^‡^	28.1 (20.0;32.3)	28.9 (22.2; 32.9)^‡^	**0.03**

Significant *p*-values are highlighted in bold

Data for pregnancies and infertility

*FSFI* Female Sexual Function Index, *HS* hidradenitis suppurativa, *IQR* interquartile range, *IVF* in vitro fertilization, *ODD* other dermatological diseases

^‡^ Indicates significant difference between the HS group and the healthy group in a post hoc analysis (FSFI-score, *p* = 0.03)

### Factors with influence on fertility

Among the participants, 44/55 (80.0%) in the HS group, 43/55 (78.2%) in the ODD group, and 45/51 (88.2%) in the HC group specified that they either had children, had been pregnant, or had attempted to get pregnant. For these participants, the number of factors known to influence fertility did not vary across the groups. Neither the age at the first attempt at conception nor the duration of the fertile period different across the three groups (see Table 3).

**Table 3:** Factors with influence on fertility

	HS (*n* = 44)	ODD (*n* = 43)	Healthy (*n* = 45)	*p* value
Age at conception attempt				
Mean (SD)	24.1 (4.3)	24.5 (4.7)	24.1 (3.6)	0.84
Fertile period (years)				
Median (IQR)	16.5 (9.0; 24.3)	10 (4.0; 16.3)	16 (6.0; 27.0)	0.08
Diseases and symptoms, before pregnancy^a^				
*n* (%)	21 (47.7)	13 (30.2)	14 (31.1)	0.63
Median (IQR)	0 (0; 1)	0 (0; 0)	0 (0; 0)	
Treatments, before pregnancy^a^				
*n* (%)	3 (6.8)	5 (11.6)	5 (11.1)	0.89
Median (IQR)	0 (0; 0)	0 (0; 0)	0 (0; 0)	
Treatments, during pregnancy^a^				
*n* (%)	2 (4.5)	0 (0)	1 (2.2)	*0.36*
Median (IQR)	0 (0; 0)	0 (0; 0)	0 (0; 0)	
Male partner comorbidities, before pregnancy^a^				
*n* (%)	2 (4.5)	0 (0)	2 (4.4)	*0.37*
Median (IQR)	0 (0; 0)	0 (0; 0)	0 (0; 0)	

Factors with influence on fertility in participants who specified to have children or had attempted to conceive

*HS* hidradenitis suppurativa, *ODD* other dermatological diseases, *IQR* interquartile range, *SD* standard deviation

^a^ See Supplementary Material for exhaustive list of factors in each category

### Univariate and multivariate analysis

In the univariate logistic regression analysis, the only factor with a statistically significant correlation with infertility was diseases and symptoms during pregnancy (OR: 3.19 (1.43–8.25), *p* = 0.01). The three adjusted models found no statistical difference in infertility among HS patients. None of the adjusted factors had a significant effect on the outcome (see Table 4).

**Table 4:** Adjusted models for infertility and HS

	Estimate	OR (95% CI)	*p* value
Model 1^a^			
Intercept	− 3.67		**0.005**
Healthy	Ref	Ref	Ref
HS	0.96	2.61 (0.85–8.65)	0.10
ODD	0.63	1.87 (0.62–6.00)	0.27
Model 2^b^			
Intercept	− 3.1		**0.02**
Healthy	Ref	Ref	Ref
HS	0.86	2.36 (0.75–7.99)	0.15
ODD	0.47	1.59 (0.50–5.32)	0.44
Model 3^c^			
Intercept	− 3.48		**0.01**
Healthy	Ref	Ref	Ref
HS	0.70	2.02 (0.37–10.75)	0.41
ODD	0.53	1.70 (0.43–6.81)	0.45

Significant *p*-values are highlighted in bold

Adjusted models for the association between infertility and HS

*HS* hidradenitis suppurativa, *ODD* other dermatological diseases, *OR* odds ratio

^a^Model 1: Adjusted for age, marital status, and BMI

^b^Model 2: Adjusted for age, marital status, BMI, smoking, alcohol and fertile period

^c^Model 3: Adjusted for age, marital status, BMI, smoking, alcohol, fertile period, FSFI score, HS severity (measured via visual analog scale (VAS)), ODD severity (measured via VAS), illnesses before pregnancy, treatments before pregnancy, treatments during pregnancy, and male partner comorbidities before pregnancy

## Discussion

In this cross-sectional study of 161 participants, we did not find a significant difference in infertility rates between the groups of HS, ODD, and HC participants. Notably, among the known factors that influence fertility, the HS group only showed a higher prevalence tobacco smoking (as measured by the median number of pack-years). Surprisingly, neither the univariate nor multivariate analyses (which adjusted for lifestyle factors, comorbidities, and medication use) identified HS as an independent factor affecting fertility.

This finding contrasts with previous research. Tzur Bitan et al.’s [17] population-based study and Adelekun et al.’s [16] survey study reported higher risk and prevalence of infertility among HS patients, respectively. However, these studies have limitations, including the absence of control for potential confounding variables that may be associated with HS, which makes it is difficult to gauge the level of causal inference provided by these studies. In our study, these confounding factors were included in the adjusted analysis and were found not to significantly influence the association between HS and infertility. Apart from smoking history before pregnancy other risk factors were not more prevalent in the HS population.

Although our study did not replicate the findings of previous literature, we acknowledge that our sample size may have been insufficient to detect a significant association. A post-hoc sample size calculation, assuming a 5% margin error, a 95% confidence level, a population size of 100.000, and an estimated proportion of 50%, indicated that a sample size of 380 participants would be needed. The inclusion of participants in this study was inhibited by the COVID-19 pandemic and the limited availability of patients in the clinic. However, our raw data did suggest a trend toward increased infertility risk among HS patients. Although this trend was not statistically significant, the higher absolute percentage of HS patients reporting infertility or childlessness suggests that a larger study might reveal a more definitive relationship.

Although our finding of lower sexual function among patients with HS is not novel [22, 23], it is interesting to consider with respect to our other findings. Reduced sexual function could potentially lead to fewer children born to HS patients. However, the absence of a significant difference in the number of children across the groups suggest that, despite lower sexual desire and function, the desire to become parents may not be diminished among patients with HS. We speculated whether the equal number of children across groups could be explained by a higher FSFI score among HS patients with children, compared to HS patients without children, but found this not to be the case. Additionally, the similar number of children across the three groups further supports this conclusion. Given that the marital status is likely to influence the likelihood of having children, and since we found no difference in marital status across the groups, it is unlikely that relationship status has confounded our results.

Compared to previous studies, the strengths of our study include the consideration of multiple confounding factors and the inclusion of a comparison group with patients with other dermatological diseases. However, limitations of this pilot study include the relatively small sample size, the reliance on self-reported data for medication use, disease severity, and the length of the fertile period, as well as the fact that all participants originated from a single center. Given the higher prevalence of HS among women in Western countries [24], our study focused exclusively on female participants. Considering that male infertility accounts for 50% of infertility cases overall, exploring an association between HS and male infertility could help clarify the role of HS as a possible contributing factor [25].

Although our current results do not support a reduced fertility among patients with HS, carrying out a larger study is warranted to fully understand whether HS acts as an independent factor influencing the fertility of female patients. Including a larger sample size, utilizing physician-confirmed diagnosis rather than self-reported data, and performing population-based or multicenter sampling strategies while still accounting for possible confounding factors, would increase statistical power providing stronger and more reliable results.

## Conclusion

Our study did not find a significant difference in infertility between patients with HS, those with other dermatological diseases, and healthy controls. This contrasts with previous studies that have reported a higher risk and prevalence of infertility among HS patients. Although our findings indicated a trend towards an increased risk of infertility in female HS patients, this trend was not statistically significant, likely due to the small sample size of our study. These results suggest that a larger, more statistical powerful study is necessary to better investigate the independent relationship between HS and infertility. Despite the limitations of our study, our findings provide valuable insights into the potential impact of HS on female fertility and highlight the need for further research in this area.

## Supplementary Information

Below is the link to the electronic supplementary material.

Supplementary file1 (DOCX 128 KB)

## Data Availability

CE Medianfar and RK Andersen had full access to all the data in the study and take responsibility for the integrity of the data and the accuracy of the data analysis. Danish health data are protected by the Danish Act on Processing of Personal Data and can only be accessed following application. Therefore, data sharing for this study is not possible.

## Abbreviations

COVID: Coronavirus disease
Dept: Department
FSFI: Female Sexual Function Index
HC: Healthy controls
HS: Hidradenitis suppurativa
IVF: In vitro fertilization
ODD: Other dermatological diseases
PCOS: Polycystic ovarian syndrome
VAS: Visual analogue scale
WHO: World Health Organization
