# Supplementary material for: Hidradenitis suppurativa and female infertility: a pilot study conducted amongst 110 dermatological patients
Source: Arch Dermatol Res. 2024 Sep 28;316(9):649. doi: 10.1007/s00403-024-03390-6 (PMC11438620; doi:10.1007/s00403-024-03390-6)
Supplement: Supplementary file 1 — Supplementary file1 (DOCX 128 KB) [file 403_2024_3390_MOESM1_ESM.docx]

**Supplementary Table 1**

**Factors associated with reduced fertility**

| **Diseases and symptoms** | **Treatments** | **Male partner diseases/symptoms** | **Male partner treatments** |
| --- | --- | --- | --- |
| High metabolism^1^ | Chemotherapy^2^ | Erectile dysfunction^3^ | Chemotherapy^4,5^ |
| Low metabolism^6^ | Radiation therapy^2^ | Varicocele^7^ | Radiation therapy^8^ |
| Hirsutism^9^ | A-vitamin treatment^10^ | Cryptorchidism^11^ | Sterilization^12^ |
| Anorexia^13^ | Methotrexate^5,14^ | Hypogonadism^15^ | Surgery on testicles^16^ |
| PCOS^17,18^ | Surgery on fallopian tubes^19^ |  | Sulfasalazine^20^ |
| Endometriosis^21^ | Surgical abortion^22^ |  | Antipsychotics^5^ |
| Amenorrhea^23^ | Sterilization^24^ |  |  |
| Metrorrhagia^23^ | Antipsychotics^25^ |  |  |
| Chlamydia^26^ |  |  |  |
| Other sexually transmitted diseases^27^ |  |  |  |

All study participants were questioned about the above-mentioned factors. They were asked to answer the relevance of these before pregnancy/before 30 years of age, when pregnant/when 30 years of age and after pregnancy/after 30 years of age, depending on their parity status.

**Supplementary Table 2**

**Univariate analysis on the association between infertility and factors with influence on fertility**

| **Univariate analysis** | | | |
| --- | --- | --- | --- |
|  | **Estimate** | **OR (CI-95%)** | **P-value** |
| **Group**   - Healthy - HS - ODD | Ref  0.77  0.53 | 2.16 (0.81 - 6.05)  1.40 (0.50 - 4.08) | 0.13  0.53 |
| **Marital status** | -0.48 | 0.62 (0.35 - 1.03) | 0.08 |
| **Smoking before pregnancy** | 0.01 | 1.01 (0.96 - 1.05) | 0.70 |
| **Alcohol consumption before pregnancy** | -0.08 | 0.92 (0.47 - 1.73) | 0.81 |
| **Fertile period** | 0.02 | 1.02 (0.97 - 1.04) | 0.89 |
| **BMI before pregnancy, per point** | 0.05 | 1.05 (0.99 - 1.12) | 0.09 |
| **Age at conception attempt, per year** | 0.06 | 1.06 (0.96 - 1.17) | 0.25 |
| **FSFI-points** | 0.04 | 1.04 (1.00 - 1.09) | 0.05 |
| **Disease and symptoms, before pregnancy, per factor**^†^ | 0.25 | 1.29 (0.80 - 2.03) | 0.28 |
| **Treatments, before pregnancy, per factor**^†^ | 0.56 | 1.75 (0.58 - 4.93) | 0.29 |
| **Male partner comorbidities, before pregnancy, per factor**^†^ | 1.18 | 3.25 (0.38 - 28.18) | 0.25 |
| **Diseases and symptoms, during pregnancy, per factor**^†^ | 1.16 | 3.19 (1.43 - 8.25) | **0.01** |
| **Treatments, during pregnancy, per factor**^†^ | 0.46 | 1.58 (0.07 - 17.05) | 0.71 |
| **VAS (ODD)** | -0.0007 | 0.99 (0.97 - 1.02) | 0.96 |
| **VAS (HS)** | 0.01 | 1.01 (1.00 - 1.03) | 0.08 |

BMI = Body Mass Index, FSFI = Female Sexual Function Index, HS = Hidradenitis Suppurativa, ODD = Other dermatological diseases, OR = Odds ratio, VAS = Visual Analogue Scale. ^†^See supplementary table 1 for exhaustive list of factors.

References

1. Mintziori, G., Kita, M., Duntas, L. & Goulis, D. G. Consequences of hyperthyroidism in male and female fertility: pathophysiology and current management. *Journal of Endocrinological Investigation* vol. 39 Preprint at https://doi.org/10.1007/s40618-016-0452-6 (2016).

2. Griffiths, M. J., Winship, A. L. & Hutt, K. J. Do cancer therapies damage the uterus and compromise fertility? *Hum Reprod Update* **26**, (2020).

3. Lotti, F. & Maggi, M. Sexual dysfunction and male infertility. *Nature Reviews Urology* vol. 15 Preprint at https://doi.org/10.1038/nrurol.2018.20 (2018).

4. Schrader, M., Heicappell, R., Müller, M., Straub, B. & Miller, K. Impact of chemotherapy on male fertility. *Onkologie* vol. 24 Preprint at https://doi.org/10.1159/000055103 (2001).

5. Buchanan, J. F. & Davis, L. J. Drug-induced infertility. *Drug Intell Clin Pharm* **18**, (1984).

6. Dosiou, C. Thyroid and Fertility: Recent Advances. *Thyroid* vol. 30 Preprint at https://doi.org/10.1089/thy.2019.0382 (2020).

7. Belker, A. M. The varicocele and male infertility. *Urologic Clinics of North America* **8**, (1981).

8. Vakalopoulos, I., Dimou, P., Anagnostou, I. & Zeginiadou, T. Impact of cancer and cancer treatment on male fertility. *Hormones* vol. 14 Preprint at https://doi.org/10.14310/horm.2002.1620 (2015).

9. Makrantonaki, E. & Zouboulis, C. C. Hyperandrogenism, adrenal dysfunction, and hirsutism. *Hautarzt* vol. 71 Preprint at https://doi.org/10.1007/s00105-020-04677-1 (2020).

10. Guillonneau, M. & Jacqz-Aigrain, E. [Teratogenic effects of vitamin A and its derivates]. *Arch Pediatr* **4**, (1997).

11. Virtanen, H. E. & Toppari, J. Cryptorchidism and Fertility. *Endocrinology and Metabolism Clinics of North America* vol. 44 Preprint at https://doi.org/10.1016/j.ecl.2015.07.013 (2015).

12. Peterson, H. B., Huber, D. H. & Belker, A. M. Vasectomy: An appraisal for the obstetrician-gynecologist. *Obstetrics and Gynecology* vol. 76 Preprint at (1990).

13. Boutari, C. *et al.* The effect of underweight on female and male reproduction. *Metabolism: Clinical and Experimental* vol. 107 Preprint at https://doi.org/10.1016/j.metabol.2020.154229 (2020).

14. Martínez Lopez, J. A., Loza, E. & Carmona, L. Systematic review on the safety of methotrexate in rheumatoid arthritis regarding the reproductive system (fertility, pregnancy, and breastfeeding). *Clinical and Experimental Rheumatology* vol. 27 Preprint at (2009).

15. Krausz, C. Male infertility: Pathogenesis and clinical diagnosis. *Best Practice and Research: Clinical Endocrinology and Metabolism* vol. 25 Preprint at https://doi.org/10.1016/j.beem.2010.08.006 (2011).

16. Berger, G. K., Smith-Harrison, L. I. & Sandlow, J. I. Sperm agglutination: Prevalence and contributory factors. *Andrologia* **51**, (2019).

17. Hoeger, K. M., Dokras, A. & Piltonen, T. Update on PCOS: Consequences, Challenges, and Guiding Treatment. *Journal of Clinical Endocrinology and Metabolism* vol. 106 Preprint at https://doi.org/10.1210/clinem/dgaa839 (2021).

18. el Hayek, S., Bitar, L., Hamdar, L. H., Mirza, F. G. & Daoud, G. Poly Cystic Ovarian Syndrome: An updated overview. *Frontiers in Physiology* vol. 7 Preprint at https://doi.org/10.3389/fphys.2016.00124 (2016).

19. Guan, J. & Watrelot, A. Fallopian tube subtle pathology. *Best Practice and Research: Clinical Obstetrics and Gynaecology* vol. 59 Preprint at https://doi.org/10.1016/j.bpobgyn.2018.12.012 (2019).

20. Bermas, B. L. Paternal safety of anti-rheumatic medications. *Best Practice and Research: Clinical Obstetrics and Gynaecology* vol. 64 Preprint at https://doi.org/10.1016/j.bpobgyn.2019.09.004 (2020).

21. Tanbo, T. & Fedorcsak, P. Endometriosis-associated infertility: aspects of pathophysiological mechanisms and treatment options. *Acta Obstetricia et Gynecologica Scandinavica* vol. 96 Preprint at https://doi.org/10.1111/aogs.13082 (2017).

22. Trichopoulos, D., Handanos, N., Danezis, J., Kalandidi, A. & Kalapothaki, V. INDUCED ABORTION AND SECONDARY INFERTILITY. *BJOG* **83**, (1976).

23. Tscherne, G. [Hormonal disorders, menstrual irregularities and future fertility]. *Gynakol Geburtshilfliche Rundsch* **43**, (2003).

24. Gizzo, S. *et al.* Female sterilization: Update on clinical efficacy, side effects and contraindications. *Minimally Invasive Therapy and Allied Technologies* vol. 23 Preprint at https://doi.org/10.3109/13645706.2014.901975 (2014).

25. Edinoff, A. N. *et al.* Hyperprolactinemia, Clinical Considerations, and Infertility in Women on Antipsychotic Medications. *Psychopharmacology bulletin* vol. 51 Preprint at (2021).

26. den Heijer, C. D. J. *et al.* Chlamydia trachomatis and the Risk of Pelvic Inflammatory Disease, Ectopic Pregnancy, and Female Infertility: A Retrospective Cohort Study among Primary Care Patients. *Clinical Infectious Diseases* **69**, (2019).

27. Tsevat, D. G., Wiesenfeld, H. C., Parks, C. & Peipert, J. F. Sexually transmitted diseases and infertility. *American Journal of Obstetrics and Gynecology* vol. 216 Preprint at https://doi.org/10.1016/j.ajog.2016.08.008 (2017).
